# Complete Genome Sequences of Newcastle Disease Virus Strains Isolated from Three Different Poultry Species in China

**DOI:** 10.1128/genomeA.00198-12

**Published:** 2013-08-15

**Authors:** Xuan Wang, Zheng Gong, Lei Zhao, Jian Wang, Gege Sun, Yali Liu, Peipei Tao, Huaikang Zhang, Shangde Li, Fei Jiang, Yuanqing Hu, Xunhai Zhang

**Affiliations:** Anhui Key Laboratory of Poultry Infectious Disease Prevention and Control, Anhui Science and Technology University, Fengyang, China

## Abstract

In 2000, three Newcastle disease virus (NDV) strains were isolated from outbreaks of infection in layers, ducklings, and geese in the same region of China during the same time period. Here, we report their complete genome sequences, which belong to the NDV genotype VIId. This discovery might provide clues as to the evolution of the NDVs of different avian origins.

## GENOME ANNOUNCEMENT

Newcastle disease (ND) is a serious disease caused by the Newcastle disease virus (NDV). The NDV genome is approximately 15.2 kb in length and encodes six structural proteins, which are arranged in the order 3′-NP–P-M-F–HN-L-5′ (1). NDV strains can be divided into class I (9 genotypes containing 15,198 nucleotides [nt] each) and class II (15 genotypes with lengths of 15,186 nt, 15,192 nt, and 15,198 nt) (2–4).

In 2000, we isolated three NDV strains from outbreaks in layers, ducks, and geese from different farms in the Anhui Province of China. In the natural outbreaks, the laying rate of the chickens decreased from 80 to 90% to 40 to 50% and the mortality rate among layers was 5%. The WF_00_C virus strain was isolated from layers that were immunized with live vaccines and boosted with inactive vaccines. The first highly pathogenic duck NDV, strain WF_00_D, was isolated in China; the incidence and mortality rates of disease caused by WF_00_D in nonimmunized meat-type ducklings were 20 to 60% and 10 to 50%, respectively. The incidence and mortality rates of disease caused by the virus strain WF_00_G in nonimmunized adult geese were 50 to 70% and 10 to 50%, respectively.

NDV virulence requires the presence of the cleavage site sequence at positions 112 to 117 of the fusion protein (5, 6), and other proteins also participate in the virus's pathogenicity (7, 8). The complete sequences of the fusion (F) genes of the three isolates were determined by reverse transcription-PCR (RT-PCR) and direct sequencing, and they have the same virulent fusion protein cleavage site sequence (^112^RRQKR↓F^117^).

The complete genomes of the three strains were amplified and cloned by using 15 pairs of oligonucleotide primers, and the sequences were determined with an ABI3730 genome sequencer from the GenScript Corporation. The complete genome sequences of the three strains were all 15,192 nucleotides in length. The three strains belong to NDV genotype VII, specifically to subgenotype VIId in class II. Compared with the vaccine strain NDV LaSota (GenBank accession no. AF077761, class II, genotype II), there is a 6-nt insert (TCCCAC) in the 5′ noncoding region (NCR) of the nucleoprotein (NP) gene. The complete genome sequences of the three NDV strains exhibit 83.0 to 83.3% homology, and the amino acid sequences of the F and hemagglutinin-neuraminidase (HN) proteins are 87.7 to 88.6% and 88.4 to 89.1% identical to those of the NDV strain LaSota, respectively. These results suggest that the WF_00_C, WF_00_D, and WF_00_G NDV strains are significantly different from the LaSota vaccine strain, potentially leading to poor vaccination protection against these strains.

Animal experiments demonstrated that these three NDV strains might induce cross-infection among chickens, ducks, and geese (9–14). These strains were all highly pathogenic in chickens and geese (12–14), and the NDV WF_00_D strain might be fatal in meat-type ducklings (13). A series of genome sequence comparisons indicated that WF_00_C and WF_00_D are 97.9% identical, WF_00_C and WF_00_G are 97.9% identical, and WF_00_D and WF_00_G are 99.3% identical. Phylogenetic analysis of the NP, phosphoprotein (P), matrix (M), HN, F, and large (L) genes in the three strains and in NDV isolates representing all of the genotypes indicated that the evolution of these six genes was isochronous. The data suggest that NDV host tropism is likely to be determined by gene mutations and multigenic control.

### Nucleotide sequence accession numbers.

The complete genome sequences have been deposited in GenBank under the accession no. FJ754271.2 (for WF_00_C), FJ754272.2 (for WF_00_D), and FJ754273.2 (for WF_00_G).
